# Dissipative dynamics of optomagnonic nonclassical features via anti-Stokes optical pulses: squeezing, blockade, anti-correlation, and entanglement

**DOI:** 10.1038/s41598-023-39822-y

**Published:** 2023-08-07

**Authors:** E. Ghasemian

**Affiliations:** Department of Electrical Engineering, Faculty of Intelligent Systems Engineering and Data Science, Persian Gulf University, Bushehr, Iran

**Keywords:** Optics and photonics, Physics

## Abstract

We propose a feasible experimental model to investigate the generation and characterization of nonclassical states in a cavity optomagnonic system consisting of a ferromagnetic YIG sphere that simultaneously supports both the magnon mode and two whispering gallery modes of optical photons. The photons undergo the magnon-induced Brillouin light scattering, which is a well-established tool for the cavity-assisted manipulations of magnons as well as magnon spintronics. At first, we derive the desired interaction Hamiltonian under the influence of the anti-Stokes scattering process and then proceed to analyze the dynamical evolution of quantum statistics of photons and magnons as well as their intermodal entanglement. The results show that both photons and magnons generally acquire some nonclassical features, e.g., the strong antibunching and anti-correlation. Interestingly, the system may experience the perfect photon and magnon blockade phenomena, simultaneously. Besides, the nonclassical features may be protected against the unwanted environmental effects for a relatively long time, especially, in the weak driving field regime and when the system is initiated with a small number of particles. However, it should be noted that some fast quantum-classical transitions may occur in-between. Although the unwanted dissipative effects plague the nonclassical features, we show that this system can be adopted to prepare optomagnonic entangled states. The generation of entangled states depends on the initial state of the system and the interaction regime. The intermodal photon-magnon entanglement may be generated and pronounced, especially, if the system is initialized with low intensity even Schrödinger cat state in the strong coupling regime. The cavity-assisted manipulation of magnons is a unique and flexible mechanism that allows an interesting test bed for investigating the interdisciplinary contexts involving quantum optics and spintronics. Moreover, such a hybrid optomagnonic system may be used to design both on-demand single-photon and single-magnon sources and may find potential applications in quantum information processing.

## Introduction

Cavity magnonics, as a new and active platform for the study of strong interactions between light and matter has emerged and developed during the past decade^1–3^. In particular, cavity magnonics deals with microwave photons in a resonant cavity interacting with magnons (i.e., collective spin excitations) in a ferrimagnetic material, e.g., yttrium iron garnet (YIG) as the most well-known ferrimagnetic insulators. Such a system exhibits some unique features and advantages, i.e., the large frequency tunability and low damping rate. Besides, magnonics systems show excellent ability to be coherently coupled with other systems including microwave or optical photons, phonons, and superconducting qubits^4–7^.

On the other hand, many notable physical phenomena have been reported in cavity magnonics such as exceptional point^8^, remote manipulation of spin current^9^, bistability^10^, cavity mediated magnon long-range coupling^11^, optical probe spin wave physics^12,13^, magnon-based quantum networks and magnon-mediated quantum gates^14^, Bose-Einstein condensation in YIG thin films^15,16^, and optical whispering-gallery modes (WGMs) in YIG spheres^5,17^. For example, the optical WGMs are particularly appealing for cavity quantum electrodynamics^18^, optomechanics^19^, and frequency combs, due to the fact that they can offer high optical quality factors and relatively small mode volumes. Experimentally, the high quality factor of YIG sphere in the telecommunication band is still challenging because of the low telecom photon absorption of YIG material and surface roughness of YIG sphere. In this line, some potentially feasible solutions have been proposed such as using other magnetic materials possessing larger Verdet constant like $\mathrm{CrBr_3}$ for enhancing the optomagnonical coupling and resorting to advanced micro- and nano-fabrication technology for reducing the surface roughness, i.e., enhancing the quality factor of YIG sphere^20,21^.

Notably, some magnetic materials are promising for future spintronics. For instance, they allow long-range information transfer^22,23^ and low-power logic^24^. Some extra unique features of magnons, e.g., long coherence times, long spin diffusion length, low energy consumption, and integration capability with traditional electronic devices are useful for “quantum magnonics” and render this field as one of the most promising research areas in spintronics^25–27^. Accordingly, a family of hybrid quantum systems has been extended by incorporating magnetic materials due to the fact that magnons can be utilized for quantum information processing. Interestingly, magnons, as information carriers with long lifetime and tunability, can remove Ohmic losses, increase memory to store information, and enhance processing capabilities^28–30^.

The potential applications of hybrid cavity magnonics systems have been extensively reported in quantum information processing and quantum sensing^21^. These hybrid optomagnonic systems demonstrate some interesting phenomena including magnon gradient memory^31^, manipulation of distant spin currents^9^, level attraction^32,33^, and nonreciprocity^34^. In particular, spintronics deals with the systems including superconducting quantum circuits coupled coherently to magnons via microwave fields in a cavity^35^. Such systems have been accomplished for generating and characterizing nonclassical magnonic states^27,36–39^, quantum thermometry protocols^40^, and for developing microwave-to-optical quantum transducers for quantum information processing^21,41^. Recently, the coherent coupling of magnons to optical photons has been experimentally demonstrated by many research groups^4,5,17,42^. Since the magnon-photon interaction gives rise to inelastic Brillouin light scattering (BLS)^43^, hence, it would be a well-established tool to study quantum magnonics^44–46^. It should be noted that magnons may be generated by optical pumps^47,48^ and more importantly optical photons can be used to probe magnons through BLS^49,50^. In addition, the WGMs coupled via an optical nanofiber can be exploited to implement the resonant structures for the enhanced BLS, and gives insights into the magnon-induced BLS^4^. Nowadays, BLS is viewed as a mature imaging tool for magnon spintronics^51,52^ and fundamental magnon studies^53,54^. Notably, the observation of Bose-Einstein condensation of magnons at room temperature in YIG thin films has been reported in^15,16^ Also, BLS experiments on YIG spheres have demonstrated a large asymmetry in the red- (Stokes) and blue-shifted (anti-Stokes) sidebands^5,17^. This asymmetry can be manipulated by controlling the polarization and wave vector of the light. Accordingly, energy can be effectively extracted from the magnons when more photons are scattered into the blue than the red-shifted sidebands. Optomagnonic scattering may be enhanced by tuning both the input and the scattered photon frequencies to the optical resonances of the cavity, i.e., via the triple resonance condition^5^. The authors in^55^ studied the magnon-induced BLS by considering a cavity optomagnonic system including the magnons and the optical WGMs. They showed that an arbitrary magnonic state (either classical or quantum) can be transferred to and stored in a distant long-lived mechanical resonator via a hybrid magnomechanical system and using optical pulses. Also, optomechanical systems with parametric coupling between optical and mechanical modes allow a promising platform for generating and manipulating nonclassical photons and phonons. For instance, the single-and two-photon blockade effects can be significantly enhanced in a nonlinear hybrid optomechanical system with optical parametric amplification^56^.

Although the strong resonant photon-magnon coupling in microwave cavities has been reported during the last two decades, the coupling in the optical regime has been demonstrated recently for the first time^57^. Hence, we are motivated to propose a theoretical scheme for generating nonclassical optomagnonic states, especially under the anti-Stokes scattering process. We demonstrate the nonclassical features of the particles, i.e., the photon and magnon antibunching and blockade phenomena under different physical conditions. Also, the cross-correlation between photons and magnons is studied in detail. The results show that the system undergoes the quantum-classical transition such that both antibunched, bunched, as well as coherent photons and magnons can be found during the interaction. Besides, it should be noted that the particles attain thermal equilibrium with their environments at the steady state regime because they are enclosed by thermal reservoirs. Furthermore, we show that the system presents strong and stable anti-correlation between the photons and magnons especially when the system is initiated with one bosonic particle, e.g., a photon or a magnon. However, the particles eventually lose their nonclassical features and become uncorrelated as the system approaches thermal equilibrium.

It is worth mentioning that, in addition to fundamental scientific significance of generation of nonclassical optomagnonic states, the photon (magnon) blockade is closely related to antibunching behavior and opens up a way to prepare a single-photon (-magnon) source for designing a single-photon gun^58^ (single-magnon emitter^59^). Also, the optomagnonic entangled states can be generated via the dissipative coupling between magnons and photons^60^. We show that the magnons and photons can be entangled by using anti-Stokes optical pulses. Particularly, the fast optical pulses are adopted to generate a transient optomagnonic entangled state^61^. In fact, the hybrid optomagnonic systems open a novel window to utilize magnon-photon entanglement as a new quantum resource for future technologies^60^.

The contents of this paper are organized as follows. In “The system and the magnon-induced BLS Hamiltonian” section, the system is introduced and its model Hamiltonian is derived, especially under the influence of the anti-Stokes scattering process. Using the Heisenberg-Langevin approach, the dissipative dynamics of the system including the analytical expressions of particle operators is investigated in “Dissipative dynamics of the system” section. In “Quantum statistics: nonclassical features, e.g., antibunching and blockade phenomena” and “Photon-magnon entanglement” sections , respectively, we analyze and discuss the dynamics of of nonclassical features, i.e., the quantum statistics of particles and intermodal photon-magnon entanglement. The experimental feasibility and applications of the system can be found in “Experimental feasibility and applications” section. Finally, we summarize the results and briefly discuss our findings and conclusions in the last section.

## The system and the magnon-induced BLS Hamiltonian

**Figure 1:**
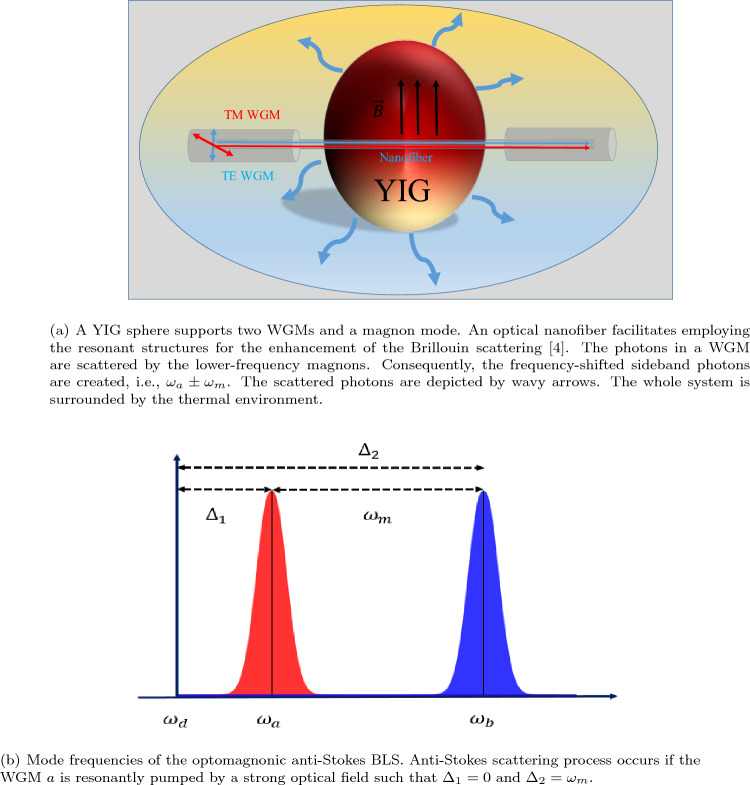
Schematic of a hybrid optomagnonic system and the corresponding mode frequencies.

Osada et al. experimentally implemented a cavity optomagnonic system such that a ferromagnetic sphere supports both photons and magnons^4^. Cavity optomagnonics with spin-orbit coupled photons demonstrates some intriguing properties, i.e., the pronounced nonreciprocity and asymmetry in the sideband signals due to the magnon-induced BLS of light^4^. The latter, e.g., magnon manipulation, has been realized by modifying the probability of the Brillouin scattering through WGMs^62^. The chirality provided by the spin dynamics in the ferromagnetic material leads not only to magnon-induced nonreciprocal Brillouin scattering but also to the creation and annihilation of magnons in a highly selective manner^4^. Since the low-frequency magnons can scatter the photons in a WGM, hence, it is referred to as the magnon-induced BLS. Such a physical condition can be achieved and governed by tuning the magnon frequency or more precisely via manipulating the strength of a static magnetic field^59^. In practice, the WGMs coupled via an optical nanofiber allow the implementation of the resonant structures to enhance the Brillouin scattering^4^. Let us consider a cavity optomagnonic system consisting of a YIG sphere that simultaneously supports two WGMs for optical photons and a magnetostatic mode for magnons as shown in Fig. 1.

The magnon-induced BLS is described by the following Hamiltonian^55^

1 $$\begin{aligned} H=H_{0}+H_{\text{int}}+H_{d}, \end{aligned}$$

where $H_{0}$ is the free Hamiltonian of the magnon and two WGMs

2 $$\begin{aligned} H_{0}= \omega _m \hat{m}^\dagger \hat{m}+ \omega _a \hat{a}^\dagger \hat{a}+ \omega _b \hat{b}^\dagger \hat{b}, \end{aligned}$$

where $\hat{m}$ ($\hat{m}^\dagger$) and $\omega _{m}$ are the annihilation (creation) operator and frequency corresponding to magnon mode *m*. Also, $\hat{S}$ ($\hat{S}^\dagger$) and $\omega _{S}$ are the annihilation (creation) operator and frequency corresponding to the WGMs denoted with $S=a,b$, respectively. Since the magnon-induced BLS is intrinsically a three-wave process, it can be described by the following interaction Hamiltonian

3 $$\begin{aligned} H_{\text{int}} = G_0 \left( \hat{m}^\dagger \hat{a} \hat{b}^\dagger + \hat{m} \hat{a}^\dagger \hat{b} \right) , \end{aligned}$$

where $G_0$ is the single-photon coupling rate. This coupling is weak due to the large frequency difference between the optical modes with respect to the magnon mode, i.e. $\omega _m \ll |\omega _a-\omega _b|$. However, the coupling strength can be considerably enhanced by intensely driving one of the WGMs. Besides, the driving Hamiltonian is given by

4 $$H_{\text{d}} = i E_a \left( \hat{a}^\dagger e^{-i\omega _{ d}t} -\hat{a} e^{i\omega _{ d}t} \right) + i E_b \left( \hat{b}^\dagger e^{-i\omega _{ d}t} -\hat{b} e^{i\omega _{ d}t} \right) ,$$

where $E_a$ ($E_b$) is the coupling strength between the WGM *a* (*b*) and the driving field of frequency $\omega _d$. Also, $E_{j}= \sqrt{P_j \kappa _j^e / \hbar \omega _d} \equiv \mathbb {K} \sqrt{P_{\text{d}}}$ denotes the coupling strength between the *j*-th WGM (with external decay rate $\kappa _j^e$) and the driving field where $\mathbb {K}=103 \mathrm{MHz/ mW^{1/2}}$ denotes the drive parameter and $P_{\text{d}}$ is the drive power^59^. Indeed, the quantities $E_j$ can be viewed as the strengths of the classical driving fields corresponding to the *j*-th WGM $j=a,b$, respectively. The model Hamiltonian (1) can be recast as the following compact form if it is taken into the rotating frame introduced by the drive frequency $\omega _{ d}$,

5 $$\begin{aligned} H_{\text{int}} = & {} \omega _m \hat{m}^\dagger \hat{m}+ \Delta _1 \hat{a}^\dagger \hat{a}+ \Delta _2 \hat{b}^\dagger \hat{b} \\ \nonumber{} & {} + \, G_0 \left( \hat{m}^\dagger \hat{a} \hat{b}^\dagger + \hat{m} \hat{a}^\dagger \hat{b} \right) \\ \nonumber{} & {} \, + i E_a \left( \hat{a}^\dagger -\hat{a} \right) + i E_b \left( \hat{b}^\dagger -\hat{b} \right) \end{aligned}$$

where $\Delta _1=\omega _a-\omega _d$ and $\Delta _2=\omega _b-\omega _d$ are the cavity-drive detunings.

## Dissipative dynamics of the system

Recently, a great deal of attention has been paid to studying hybrid magnonic systems. There exist various methods for dealing with the dynamical evolution of such systems, e.g., the well-known quantum master equation, and the Heisenberg-Langevin approaches. The former usually results in a complicated set of differential equations, thus finding the analytical solution of master equations may be cumbersome or even impossible in some cases. Hence, many authors have resorted to the numerical methods and/or investigated the steady state of their considered system. In particular, some studies have focused on the nonclassical features (e.g., antibunching, blockade effect, entanglement) of magnonic systems at steady state regime^59,61,63^. For instance, kheirabady et al. numerically investigated the steady state quantum statistics of a hybrid optomechanical-ferromagnet system using quatum master approach^64^. Also, the steady-state magnon-photon entanglement in a hybrid magnet-cavity system was studied by Li et al.^65^. In contrast, here, we proceed to use the latter i.e., the Heisenberg-Langevin approach, and find some analytical expressions corresponding to the operators of photonic and magnonic subsystems. Then, we investigate the nonclassical features of photonic and magnonic states both analytically and numerically. In particular, we show how to achieve both photon and magnon antibunching and blockade phenomena as well as the photon-magnon entanglement. Besides, we follow the behavior of the mentioned effects at the steady state regime.

### Scattering process

BLS is an established technique to study magnons. In this line, the authors in^66^ theoretically studied the inelastic scattering of photons by a magnetic sphere that supports WGMs in a plane normal to the magnetization. In fact, magnons with low angular momenta scatter the light in the forward direction with a pronounced asymmetry in the Stokes and the anti-Stokes scattering strength. The analysis of the Brillouin scattering strength for the TE and TM input signals can be found in^4^. Some magnetic systems show such an asymmetry due to the interference of photons^67–69^. The ellipticity of the spin waves caused by magnetic anisotropies is a possible source of such asymmetry^70^. Besides, the Stoke and anti-Stokes asymmetry in WGM cavities may occur due to the partial elliptical polarization of WGMs^4,17^ or the interplay of birefringence and conservation laws^5^. Importantly, such an asymmetry affects the generation of macroscopic quantum states of magnons in optomagnonics^20,71,72^.

**Figure 2:**
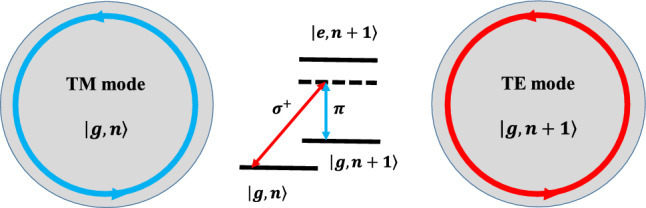
BLS process in an optomagnonic system. Generally, the transition $\left| g,n\right\rangle \rightarrow \left| g,n+1\right\rangle$ occurs via the excited state $\left| e,n+1\right\rangle$ by creating a magnon and a down-converted sideband photon with $\pi$ polarization in the TE mode^4^. In the Stokes scattering process, the pump (TM-polarized) photons convert into lower-frequency sideband (TE-polarized) photons by creating magnon excitations. By contrast, the anti-Stokes scattering occurs if the pump (TE-polarized) photons convert into higher-frequency (TM-polarized) photons by annihilating magnons^55^.

Here, it would be of interest to clarify how the selection rule affects the Brillouin scattering. Assume the states $\left| g,n\right\rangle$ and $\left| e,n\right\rangle$, respectively denote the electronic ground and excited states of the optical transition, $\left| g\right\rangle \leftrightarrow \left| e\right\rangle$, and *n* is the number of magnons in the Kittel mode, $\left| n\right\rangle$. When the input photons are in the TM mode, the light in the resonator is $\sigma ^+$ polarized as shown in Fig. 2. Hence, the transition $\left| g,n\right\rangle \rightarrow \left| g,n+1\right\rangle$ takes place via the excited state $\left| e,n+1\right\rangle$ by creating a magnon and a down-converted sideband photon with $\pi$ polarization in the TE mode. In contrast, if the input photons are in the TE mode, the light in the resonator is $\pi$ polarized. Therefore, the annihilation of a magnon and the creation of an up-converted blue-sideband photon with $\sigma ^+$ polarization in the TM mode may be expected in the reverse process. As the laser wavelength is far detuned from the transition, therefore, the excited state $\left| e\right\rangle$ is only virtually populated. Further details can be found in^4^. Note that the dominant optical transition is considered to be the spin- and parity-allowed ${ ^6S(3d^52p^6) \leftrightarrow ^6P(3d^62p^5)}$ charge transfer transition in YIG^4,73^. Here, we want to focus on the effects of the anti-Stokes scattering process which is responsible for the optomagnonical state-swap interaction^61^.

### Anti-Stokes scattering process

Due to the asymmetry nature of the BLS, both Stokes and anti-Stokes scattering events may occur corresponding to the process of creating and annihilating magnons, respectively. Such a mechanism has been applied for the manipulation of magnons and adapted for preparing nonclassical states of magnons. In particular, the anti-Stokes scattering occurs if a TE-polarized photon converts into a TM-polarized photon via annihilating magnons^74^. Let us focus on the latter case and consider the case in which the WGM *a* is resonantly pumped by a strong optical field such that $\Delta _1=0$ and $\Delta _2=\omega _m$. The mode frequencies corresponding to such a physical process are shown in Fig. 1b. Therefore, the system’s model Hamiltonian in the interaction picture reads as

6 $$\begin{aligned} H_{\text{int}}^\text{AS}= & {} \omega \left( \hat{m}^\dagger \hat{m}+ \hat{b}^\dagger \hat{b} \right) + G \left( \hat{m}^\dagger \hat{b}+ \hat{m} \hat{b}^\dagger \right) + i E_a \left( \hat{a}^\dagger -\hat{a} \right) + i E_b \left( \hat{b}^\dagger -\hat{b} \right) , \end{aligned}$$

where $G= G_0 \alpha$ is the effective photon-magnon coupling strength such that $\alpha _a=2E_a/\kappa _a$ and $\kappa _a$ denotes the linewidth (FWHM) of the WGM *a*. Note that $N_a=|\alpha _a|^2$ is the corresponding intracavity photon number. This Hamiltonian describes the interaction between the WGM *b* and the magnon mode *m* that can be used to read out the magnon state by measuring the created anti-Stokes optical field *b*. In practice, the magnonic state can be read out from the output optical field via the mechanical transduction^61^. The corresponding quantum Langevin equations by taking into account the dissipation process and input noise for each mode are given by

7 $$\begin{aligned} \dot{\hat{b}}(t)= & {} -u \hat{b}(t)-iG \hat{m}(t)+E_b+\hat{F}_b(t),\\ \nonumber \dot{\hat{m}}(t)= & {} - v \hat{m}(t)-iG \hat{b}(t)+\hat{F}_m(t), \end{aligned}$$

which is a coupled set of ordinary differential equations. In Eq. (7), we have set $u=i\omega +\kappa _b$ and $v=i\omega +\kappa _m$ where $\kappa _b$ and $\kappa _m$ being the linewidths and $\hat{F}_b(t)=-i \sum _k g_k \hat{b}_k (0) e^{-i\omega _k t}$ and $\hat{F}_m(t)=-i \sum _k g_k \hat{m}_k (0) e^{-i\omega _k t}$ denote the noise operators corresponding to the WGM mode *b* and the magnon mode *m*, respectively. Let us take the Laplace transform of the above equations and define $B(s)=L [\hat{b}(t)]$, $M(s)=L [\hat{m}(t)]$, $F_b(s)=L [\hat{F}_b(t)]$ and $F_m(s)=L [\hat{F}_m(t)]$. Therefore, we arrive at

8 $$\begin{aligned} B(s)= & {} \frac{\hat{b}(0)-iG M(s)+E_b+F_b(s)}{s+u}, \end{aligned}$$

9 $$\begin{aligned} M(s)= & {} \frac{\hat{m}(0)-iG B(t)+F_m(s)}{s+v}. \end{aligned}$$

Upon inserting Eq. (9) into Eq. (8) or vice-versa and performing some straightforward calculations we obtain

10 $$\begin{aligned} B(s)= & {} \frac{(s+u) \left( \hat{b}(0)+E_b+F_b(s) \right) }{(s+a_1)(s+a_2)} -\frac{iG \left( \hat{m}(0)+F_m(s) \right) }{(s+a_1)(s+a_2)}, \end{aligned}$$

11 $$\begin{aligned} M(s)= & {} \frac{(s+v)(\hat{m}(0)+F_m(s))}{(s+a_1)(s+a_2)} -\frac{iG(\hat{b}(0)+F_b(s))}{(s+a_1)(s+a_2)}, \end{aligned}$$

where $a_1=i\omega +\kappa _b+iG$ and $a_2=i\omega +\kappa _m-iG$. In what follows, we set $\kappa _b=\kappa _m=\Gamma$ for the sake of simple analysis. Taking the inverse Laplace transform of Eq. (10) results in the time-dependent operator corresponding to the WGM *b*

12 $$\begin{aligned} \hat{b}(t)= & {} e^{-(i\omega +\Gamma )t}[(\hat{b}(0)+E_b)\cos Gt-i \hat{m}(0) \sin Gt]+ \hat{\mathcal {B}}_{\text{B}}(t)+ \hat{\mathcal {M}}_{\text{B}}(t), \end{aligned}$$

where

13 $$\begin{aligned} \hat{\mathcal {B}}_{\text{B}}(t)= & {} -i \sum _k g_k \hat{b}_k(0) B(t),\\ \nonumber \hat{\mathcal {M}}_{\text{B}}(t)= & {} - G \sum _k g_k \hat{m}_k(0) M(t). \end{aligned}$$

Also, the inverse Laplace transform of Eq. (11) gives the dynamical evolution of the magnon operator

14 $$\begin{aligned} \hat{m}(t)= & {} e^{-(i\omega +\Gamma )t} \left[ \hat{m}(0)\cos Gt-i \left( \hat{b}(0)+E_b \right) \sin Gt \right] + \hat{\mathcal {B}}_{\text{m}}(t)+ \hat{\mathcal {M}}_{\text{m}}(t), \end{aligned}$$

where

15 $$\begin{aligned} \hat{\mathcal {B}}_{\text{m}}(t)= & {} -i \sum _k g_k \hat{m}_k(0) B(t), \\ \nonumber \hat{\mathcal {M}}_{\text{m}}(t)= & {} - G \sum _k g_k \hat{b}_k(0) M(t), \end{aligned}$$

where we have defined the following relations

16 $$\begin{aligned} B(t)= & {} \frac{e^{-(i\omega +\Gamma +iG)t}}{2[i\omega _k-(i\omega +\Gamma +iG)]}+\frac{e^{-(i\omega +\Gamma -iG)t}}{2[i\omega _k-(i\omega +\Gamma -iG)]} \\ \nonumber{} & {} + \frac{(i\omega +\Gamma -i\omega _k)e^{-i \omega _k t}}{(i\omega +\Gamma +iG-i\omega _k)(i\omega +\Gamma -iG-i\omega _k)}, \end{aligned}$$

17 $$\begin{aligned} M(t)= & {} \frac{e^{-(i\omega +\Gamma +iG)t}}{-2iG[i\omega _k-(i\omega +\Gamma +iG)]}+\frac{e^{-(i\omega +\Gamma -iG)t}}{2iG[i\omega _k-(i\omega +\Gamma -iG)]} \\ \nonumber{} & {} + \frac{e^{-i \omega _k t}}{(i\omega +\Gamma +iG-i\omega _k)(i\omega +\Gamma -iG-i\omega _k)}. \end{aligned}$$

It is worth noting that the operators of WGM *b* and the magnon mode *m* have similar time-dependent expressions, especially for $E_b=0$. One can check that $\hat{m}(t)$ can be obtained from $\hat{b}(t)$ by replacing $\hat{m}(0)$ with $\hat{b}(0)$ and vice-versa which is due to the symmetry of the Hamiltonian (6) with respect to both WGM *b* and the magnon mode *m*. In what follows we set $\hat{b} \equiv \hat{b}(0)$ and $\hat{m} \equiv \hat{b}(0)$ for convinience.

## Quantum statistics: nonclassical features, e.g., antibunching and blockade phenomena

In this section, we want to evaluate the quantum statistics of the particles, i.e., photons and magnons. At first, we proceed to study the auto-correlation of these particles and then investigate their cross-correlation.

### Auto-correlation

In order to find under what conditions the nonclassical features of the photons and magnons, i.e., antibunching and blockade phenomena can be observed, let us begin with their auto-correlations. The zero-delay second-order auto-correlation function yields the auto-correlation among the bosons, i.e., photons or magnons^75^

18 $$\begin{aligned} g_x^{(2)}(0)= & {} \frac{\left\langle \hat{x}^\dagger (t)\hat{x}^\dagger (t)\hat{x}(t)\hat{x}(t)\right\rangle }{\left\langle \hat{x}^\dagger (t)\hat{x}(t)\right\rangle ^2}, \qquad x=b,m. \end{aligned}$$

The quantum state or more precisely the nonclassical features of these bosonic particles can be identified based on the value of $g^{(2)}(0)$^75,76^. Generally, three different cases are characterized as follows.

- $g^{(2)}(0)>1$ implies that the particles are bunched without nonclassical features, i.e., the particle distribution is super-Poissonian.
- $g^{(2)}(0)=1$ indicates that the particles show Poissonian distribution with coherent properties.
- $g^{(2)}(0)<1$ demonstrates the nonclassical features, i.e., the antibunched particles with the sub-Poissonian distribution. In this case, the particles possess pure quantum-mechanical states that cannot be captured by classical statistics^77^. Besides, $g^{(2)}(0) = 0$ indicates a perfect particle blockade effect, i.e., a signature of a single-magnon (single-photon) source. In what follows, we theoretically demonstrate the feasibility of using our hybrid optomagnonic system for preparing the single-particle sources based on blockade effects. Physically, the magnon (photon) blockade phenomenon implies that the excitation of multiple magnons (photons) is blocked at the same time^59^. Besides its fundamental scientific significance, the magnon blockade effect is crucial for the exploration of magnon at the quantum level and opens up a pathway for designing a single-magnon emitter^78–80^. Now, we derive the analytical expressions corresponding to the auto-correlation functions of photons in the WGM *b* and the magnon mode *m*. The auto-correlation function of the WGM *b* can be obtained as
  19 $$\begin{aligned} g_{ b}^{(2)}(0)= & {} \frac{W_1(t)}{W_2(t)}, \end{aligned}$$

where

20 $$\begin{aligned} W_1(t)= & {} e^{-4\Gamma t} \big [\left\langle ({b^\dagger }^2 b^2 + 4 E_b^2 b^\dagger b + E_b^4)\right\rangle \cos ^4 G t \\ \nonumber{} & {} + \, 4 (b^\dagger b+E_b^2) m^\dagger m \cos ^2 G t \sin ^2 G t + {m^\dagger }^2 m^2 \sin ^4 G t \big ] \\ \nonumber{} & {} + \, 4 e^{-2\Gamma t} \big [ \left\langle (b^\dagger b+E_b^2) \cos ^2 G t +m^\dagger m \sin ^2 G t \right\rangle \big ] ( \left\langle \hat{\mathcal {B}}^\dagger _{\text{B}}(t) \hat{\mathcal {B}}_{\text{B}}(t) \right\rangle +\left\langle \hat{\mathcal {M}}^\dagger _{\text{B}}(t) \hat{\mathcal {M}}_{\text{B}}(t) \right\rangle ) \\ \nonumber{} & {} + \, 2\big [ \left\langle \hat{\mathcal {B}}^\dagger _{\text{B}}(t) \hat{\mathcal {B}}_{\text{B}}(t) \right\rangle ^2+2 \left\langle \hat{\mathcal {B}}^\dagger _{\text{B}}(t) \hat{\mathcal {B}}_{\text{B}}(t) \right\rangle \left\langle \hat{\mathcal {M}}^\dagger _{\text{B}}(t) \hat{\mathcal {M}}_{\text{B}}(t) \right\rangle + \left\langle \hat{\mathcal {M}}^\dagger _{\text{B}}(t) \hat{\mathcal {M}}_{\text{B}}(t) \right\rangle ^2 \big ], \end{aligned}$$

and

21 $$\begin{aligned} W_2(t)= & {} e^{-2\Gamma t} \big [ \big (b^\dagger b+E_b^2 \big )\cos ^2 G t + m^\dagger m \sin ^2 G t \big ) \big ] \\ \nonumber{} & {} + \, \left\langle \hat{\mathcal {B}}^\dagger _{\text{B}}(t) \hat{\mathcal {B}}_{\text{B}}(t) \right\rangle + \left\langle \hat{\mathcal {M}}^\dagger _{\text{B}}(t) \hat{\mathcal {M}}_{\text{B}}(t) \right\rangle . \end{aligned}$$

Similarly, the auto-correlation function of the magnon mode *m* read as

22 $$\begin{aligned} g_m^{(2)}(0)= & {} \frac{R_1(t)}{R_2(t)}, \end{aligned}$$

where

23 $$\begin{aligned} R_1(t)= & {} e^{-4\Gamma t} \left[ \left\langle {m^\dagger }^2 m^2 \right\rangle \cos ^4 G t \right. \\ \nonumber{} & {} + \, \left. 4 m^\dagger m \left( b^\dagger b+E_b^2 \right) \cos ^2 G t \sin ^2 G t + \left( {b^\dagger }^2 b^2+4 E_b^2 b {b^\dagger }+E_b^4 \right) \sin ^4 G t \right] \\ \nonumber{} & {} + \, 4 e^{-2\Gamma t} \left[ \left\langle m^\dagger m \cos ^2 G t + \left( b^\dagger b+E_b^2 \right) \sin ^2 G t \right\rangle \right] \left( \left\langle \hat{\mathcal {B}}_{\text{m}}(t) \hat{\mathcal {B}}^\dagger _{\text{m}}(t) \right\rangle +\left\langle \hat{\mathcal {M}}^\dagger _{\text{m}}(t) \hat{\mathcal {M}}_{\text{m}}(t) \right\rangle \right) \\ \nonumber{} & {} + \, 2\left[ \left\langle \hat{\mathcal {B}}_{\text{m}}(t) \hat{\mathcal {B}}^\dagger _{\text{m}}(t) \right\rangle ^2+2 \left\langle \hat{\mathcal {B}}_{\text{m}}(t) \hat{\mathcal {B}}^\dagger _{\text{m}}(t) \right\rangle \left\langle \hat{\mathcal {M}}^\dagger _{\text{m}}(t) \hat{\mathcal {M}}_{\text{m}}(t) \right\rangle + \left\langle \hat{\mathcal {M}}^\dagger _{\text{m}}(t) \hat{\mathcal {M}}_{\text{m}}(t) \right\rangle ^2 \right] \end{aligned}$$

and

24 $$\begin{aligned} R_2(t)= & {} e^{-2\Gamma t} \big [ m^\dagger m\cos ^2 G t + \big ( b^\dagger b+E_b^2 \big ) \sin ^2 G t \big ) \big ] \\ \nonumber{} & {} + \left\langle \hat{\mathcal {B}}_{\text{m}}(t) \hat{\mathcal {B}}^\dagger _{\text{m}}(t) \right\rangle + \left\langle \hat{\mathcal {M}}^\dagger _{\text{m}}(t) \hat{\mathcal {M}}_{\text{m}}(t) \right\rangle . \end{aligned}$$

Since various physical contexts are characterized by different forms of environmental spectra, which in turn identifies how fast the system decays. Therefore, in order to numerically analyze the auto-correlation functions, one should define the environments’ spectra. Here, we consider the same parametrized spectral density function for both photons and magnons as^81^

25 $$\begin{aligned} J(\omega )= \eta \omega ^s \omega _c^{1-s} e^{-\omega /\omega _c} \end{aligned}$$

where $\eta$ stands for the so-called Kondo parameter which denotes the dimensionless strength of the system-bath coupling and $\omega _c$ is the cut-off frequency of the environment (bath). Typically, three types of environments are identified, i.e., the super-Ohmic ($s>1$), Ohmic ($s=1$), and sub-Ohmic ($s<1$) environments. When the bath spectral density changes from super-Ohmic to sub-Ohmic, a notable transition from coherent to incoherent dynamics takes place, especially in the spin-boson model. Therefore, a localization transition may be expected at high values of $\eta$ in the limit $\omega _c \rightarrow \infty$ and low temperature $T \rightarrow 0$, for the sub-Ohmic and Ohmic cases^82^. There exists no such a transition for the super-Ohmic dissipation. In the sub-Ohmic regime, the dissipative dynamics strongly depends on the initial preparation, particularly, at low temperatures. In the Ohmic regime, the reported Kondo parameter reads as $0.5< \eta < 1$^83^. Generally speaking, the lower values of the Kondo parameter may be expected for the super-Ohmic dissipation process, e.g., $\eta =0.1$^84^. It is worth noting that ferromagnetic and antiferromagnetic materials in noisy environments may qualitatively present similar behavior in the Ohmic, but they show fundamentally different behaviors in the super-Ohmic environments. In particular, in super-Ohmic noisy environments, the antiferromagnet (in contrast to the ferromagnet) shows a magnon decay rate that goes to zero at the largest values of the wave vectors in the Brillouin zone. Hence, it would be of fundamental interest for magnonics and quantum information transport^81^. Let us proceed to compute the expectation values of the auto-correlation functions corresponding to the time evolution of the WGM operator *b* under the influence of the anti-Stokes scattering process. Considering a super-Ohmic spectral density function ($s=3$), one can obtain the system-bath expectation values as follows (further details can be found in the Appendix)

26 $$\begin{aligned} \left\langle \hat{\mathcal {B}}^\dagger _{\text{B}}(t)\hat{\mathcal {B}}_{\text{B}}(t)\right\rangle= & {} \eta \omega _c^{-2} \chi ^2(t) (k_{\text{B}}T)^4 \Psi \left( {3,\frac{p+1}{p}}\right) , \end{aligned}$$

where we have set the integer $p= \omega _c/ k_{\text{B}}T$ for convenience. Also, we have defined

27 $$\begin{aligned} \chi (t)= & {} \frac{-e^{-\Gamma t} (\Gamma \cos Gt - G \sin Gt)+ \Gamma }{G^2+\Gamma ^2}. \end{aligned}$$

With the same approach, one can obtain the following relations

28 $$\begin{aligned} \left\langle \hat{\mathcal {M}}^\dagger _{\text{B}}(t)\hat{\mathcal {M}}_{\text{B}}(t)\right\rangle = \eta \omega _c^{-2} \Theta ^2 (t) (k_{\text{B}}T)^4 \Psi \left( {3,\frac{p+1}{p}}\right) , \end{aligned}$$

where

29 $$\begin{aligned} \Theta (t)=\frac{-e^{-\Gamma t} (G \cos Gt + \Gamma \sin Gt)+ G}{G^2+\Gamma ^2}. \end{aligned}$$

Other expectation values can be obtained straightforwardly.

### Cross-corrlation

Another interesting statistical property of a hybrid optomagnonic system is the cross-correlation between its components. This quantity is a measure of the coincidence counting of the photons and magnons at time *t*. In practice, it would be measured by a Hanbury-Brown and Twiss type of experiment for interference between two different beams (one for each mode)^75,85^. The cross-correlation function can be obtained as

30 $$\begin{aligned} g_{mb}^{(2)}(0)= & {} \frac{\left\langle \hat{m}^\dagger (t)\hat{b}^\dagger (t)\hat{b}(t)\hat{m}(t)\right\rangle }{\left\langle \hat{b}^\dagger (t)\hat{b}(t)\right\rangle \left\langle \hat{m}^\dagger (t)\hat{m}(t)\right\rangle }. \end{aligned}$$

Based on the value of this quantity the particles are characterized such that $g_{mb}^{(2)}(0)=1$ implies that the two modes (photons and magnons) are uncorrelated. Also, $g_{mb}^{(2)}(0)>1$ demonstrates that the photons and magnons are correlated. By contrast, $g_{mb}^{(2)}(0)<1$ indicates that the photonic mode and the magnonic mode are anit-correlated. The latter implies that there is no tendency for them to appear simultaneously. Anti-correlations between pairs of modes have been observed in other contexts such as light scattering by non-spherical particles^86^ and also in ring lasers^87^ when one mode is coherent and the other mode is chaotic above the threshold. Correlation effects between the two modes are also encountered in quantum optic systems^88^. The analytical expression of the cross-correlation between the photons and magnons in our considered system reads as

31 $$\begin{aligned} g_{mb}^{(2)}(0) = \frac{ D(t) }{W_2(t) R_2(t)}, \end{aligned}$$

where

32 $$\begin{aligned} D(t)= & {} \left\langle D_1(t)\right\rangle +\left\langle D_2(t)\right\rangle +D_3(t), \\ \nonumber D_1(t)= & {} e^{-4\Gamma t} \left\{ \hat{m}^\dagger \hat{m} \left( \hat{b}^\dagger \hat{b}+E_b^2 \right) \cos ^2 2Gt \right. \\ \nonumber{} & {} \left. + \left[ \hat{b}^\dagger \hat{b}^\dagger +2E_b \hat{b}^\dagger +E_b^2+\hat{m}^\dagger \hat{m}^\dagger \right] \times \left[ \hat{b} \hat{b} +2E_b \hat{b}+E_b^2+\hat{m} \hat{m} \right] \sin ^2 Gt \cos ^2 Gt \right\} , \\ \nonumber D_2(t)= & {} e^{-2\Gamma t} \left\{ [(\hat{b}^\dagger \hat{b}+E_b^2) \cos ^2 Gt + \hat{m}^\dagger \hat{m} \sin ^2 Gt ] \times \left( \hat{\mathcal {B}}^\dagger _{\text{m}}(t) \hat{\mathcal {B}}_{\text{m}}(t) + \hat{\mathcal {M}}^\dagger _{\text{m}}(t) \hat{\mathcal {M}}_{\text{m}}(t) \right) \right. \\ \nonumber{} & {} + \, \left. \left[ \hat{m}^\dagger \hat{m} \cos ^2 Gt + \left( \hat{b}^\dagger \hat{b}+E_b^2 \right) \sin ^2 Gt \right] \times \left( \hat{\mathcal {B}}^\dagger _{\text{B}}(t) \hat{\mathcal {B}}_{\text{B}}(t) + \hat{\mathcal {M}}^\dagger _{\text{B}}(t) \hat{\mathcal {M}}_{\text{B}}(t) \right) \right\} \end{aligned}$$

and

33 $$\begin{aligned} D_3(t)= & {} \left\langle \hat{\mathcal {M}}^\dagger _{\text{B}}(t) \hat{\mathcal {M}}_{\text{B}}(t)\right\rangle \left\langle \hat{\mathcal {M}}^\dagger _{\text{m}}(t) \hat{\mathcal {M}}_{\text{m}}(t)\right\rangle + \left\langle \hat{\mathcal {B}}^\dagger _{\text{B}}(t) \hat{\mathcal {B}}_{\text{B}}(t)\right\rangle \left\langle \hat{\mathcal {B}}^\dagger _{\text{m}}(t) \hat{\mathcal {B}}_{\text{m}}(t)\right\rangle \\ \nonumber{} & {} + \, \left\langle \hat{\mathcal {B}}^\dagger _{\text{B}}(t) \hat{\mathcal {B}}_{\text{B}}(t)\right\rangle \left\langle \hat{\mathcal {M}}^\dagger _{\text{m}}(t) \hat{\mathcal {M}}_{\text{m}}(t)\right\rangle + \left\langle \hat{\mathcal {M}}^\dagger _{\text{B}}(t) \hat{\mathcal {M}}_{\text{B}}(t)\right\rangle \left\langle \hat{\mathcal {B}}^\dagger _{\text{m}}(t) \hat{\mathcal {B}}_{\text{m}}(t)\right\rangle \end{aligned}$$

where in Eq. (33) we have used the following decorrelation approximation^89^

34 $$\begin{aligned} \left\langle ABCB\right\rangle \simeq \left\langle AB\right\rangle \left\langle CD\right\rangle +\left\langle AC\right\rangle \left\langle BD\right\rangle +\left\langle AD\right\rangle \left\langle BC\right\rangle . \end{aligned}$$

### Numerical results

Let us now numerically examine the time evolution of quantum statistics of the components of the system, i.e., photons and magnons. To do so, we try to use the experimentally feasible parameters reported in the relevant literature. For instance, the damping rate, $\Gamma \simeq 1.4$ MHz^59^, the scaled driving field strength, $E_a= 2\pi \times 0.1$ MHz ($P_d=0.037\, \upmu$W^59,61^), and the effective magnon-photon coupling strength, $G=2\pi \times 3.2$ MHz at low temperatures $T=10$ mK^65^ have been used in numerical simulations. Here, we examine the nonclassical features of particles by considering long-range parameters. However, a specific parameter range may be selected to achieve nonclassical features depending on the structure of the system and its components. Also, it should be noted that the cut off frequency strongly depends on the temperature. For super-Ohmic dissipation, this parameter may take different values $\omega _c=100, 500 \, \mathrm{cm^{-1}}$ even at zero temperature^81^. So it is reasonable to consider $\omega _c \gg k_b T$ for numerical calculations. Besides, generally, the spectral density of the environment in the super-Ohmic regime ($s>1$) reads as $J(\omega ) \propto \omega ^s$ for $\omega \ll \omega _c$. For instance $\hbar \omega _c \simeq 1000 \, k_b T$ can be found in^90^.

In the following, we discuss the dependence of the nonclassical features on the system parameters, i.e., the driving strength, the effective photon-magnon coupling strength, the initial particles’ numbers, etc. Also, we demonstrate how the system must be initiated to achieve strong anti-bunching and blockade phenomena.

#### Photon statistics

**Figure 3:**
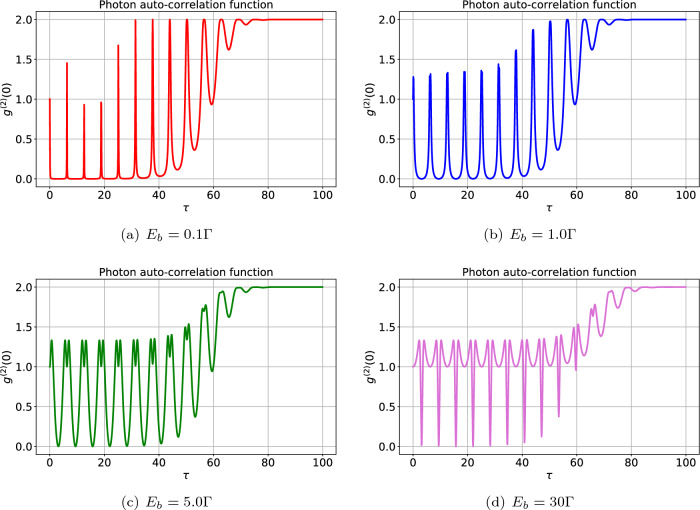
The effect of driving field strength $(E_b)$ on the photon statistics against the scaled time $\tau =\Gamma t$ for $n=0$, $m=1$ and $G=5\Gamma$ with $\Gamma =1.0 \gamma$. Note that we have set $\gamma =2 \pi \times 1.0$ MHz to scale all the frequencies throughout the article. The other parameters are chosen as $\omega _c=100\Gamma$, $\eta =0.1$, $k_{\text{B}}T=0.001 \omega _c$ for all numerical results. The results show that the desired nonclassical features such as the photon antibunching ($g_b^{(2)} (0)<1$) and photon blockade ($g^{(2)} (0) \rightarrow 0$) take place during the interaction. However, the photons become thermal particles ($g_b^{(2)} (0) =2$) as the system approches steady state.

Figure 3 shows the dynamical evolution of $g_b^{(2)} (0)$ for different values of the driving field strength corresponding to the WGM *b* indicated by $E_b$. One can see that the photons show coherent properties in the beginning of the interaction, i.e., $g_b^{(2)} (0)=1$ because there is no photon in the WGM *b*. The initial coherent feature is contributed to the classical driving field. As time passes both photons and magnons are generated and then the photons scattered by the low-frequency magnons. The scattered photons may go into the WGM (via the so-called triple-resonance condition)^55^ and acquire nonclassical properties and finally attain thermal equilibrium with the thermal environment. In particular, the time evolution of photon statistics with relatively weak driving field $E_b=0.1 \Gamma$, shown in Fig. 3a, demonstrates a periodic manner after the onset of interaction such that at most times the perfect photon blockade takes place, i.e., $g_b^{(2)} (0)=0$. However, some fast transitions from pure quantum to classical properties occur at some moments of time, i.e., $g_b^{(2)} (0)=0 \rightarrow g_b^{(2)} (0)>1$. Naturally, the photons lose their nonclassical features as they approach thermal equilibrium in the steady state regime. Note that $\lim _{t \rightarrow \infty } g_b^{(2)} (0)=2$ is a signature of the appearence of thermal photons. By increasing the strength of the driving field, e.g., $E_b=1.0 \Gamma$, the same behavior can be found in Fig. 3b. In this case, the perfect photon blockade phenomenon occurs in shorter time intervals with respect to the previous case. In other words, the effect of photon blockade is limited by growing the strength of the driving field. Accordingly, Fig. 3c,d show that classical photons prevail by further increasing the strength of the driving field such that for $E_b=30 \Gamma$ the perfect photon blockade effect can be observed just at some moments of time. In other words, sub-Poissonian (super-Poissonian) photons may be found in longer times by considering a relatively weak (strong) driving field.

**Figure 4:**
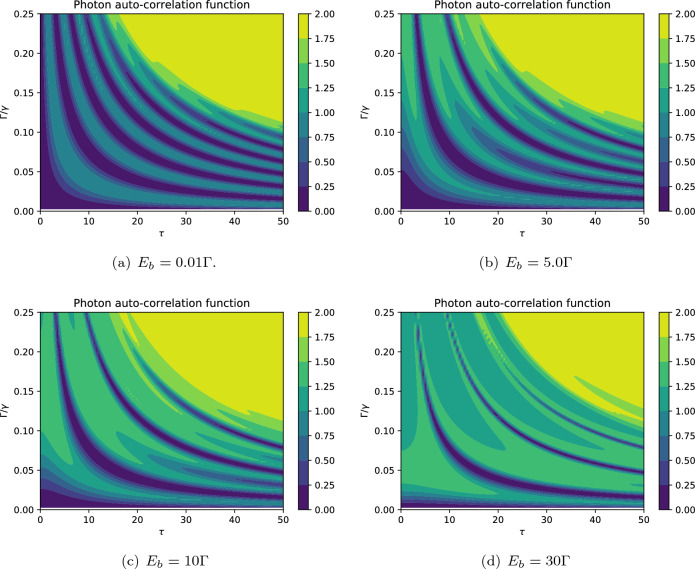
The effect of driving field strength on the dynamics of photons’ auto-correlation function against the scaled time $\tau =\Gamma t$ for $n=1$, $m=1$, $G=2\Gamma$. The other parameters are the same as in Fig. 3. The desired nonclassical features such as the photon antibunching ($g_b^{(2)} (0)<1$) and photon blockade ($g_b^{(2)} (0) \rightarrow 0$) can be realized.

The contours of photon auto-correlation function are plotted in Fig. 4. The patterns indicate that the values of the auto-correlation function separate the nonclassical regime $g_b^{(2)} (0)<1$ from the classical regime $g_b^{(2)} (0)>1$. Also, the photon blockade effect can be observed in all cases, especially when $g_b^{(2)} (0) \rightarrow 0$. Generally, the results presented in Fig. 4 demonstrate that the generation of nonclassical photons strongly depends on the driving field strength $E_b$ such that by increasing this quantity the photons get more classical features as previously described in Fig. 3. In particular, a simple comparison between Fig. 4a,d reveals that the photon blockade phenomenon is more plausible in the weak driving field regime.

Besides, the photon statistics has a periodic behavior with respect to both scaled time and the damping rate $\Gamma$. In fact, at the onset of the interaction, the photnos show sub-Poissonian statistics for small values of damping rate. During the interaction (at the moderate time), the photon statistics periodically changes from sub-Poissonian to super-Poissonian, i.e., implying the quantum-classical transition. Also, the coherent (Poissonian) photons may be observed during the interaction, especially by applying a strong driving field. Note that the quantum-classical transitions become faster for larger values of damping rates. Indeed, the photons sooner attain thermal equilibrium, i.e., steady state regime, by increasing the amount of damping rate.

The so-called photon antibunching may arise from various mechanisms, and implies that finding two or more photons is not plausible at the same time. The potential applications of single-photon states for optical processing of quantum information have been extensively discussed in relevant literature^58,91^. A reliable and bright source of single-photons would be of interest for spectroscopy and quantum optics. One of the essential features of a single-photon emitter is that they never emit two or more photons (with the same wavelength) simultaneously and consequently present strong antibunching. The single-photon sources (photon guns) have been realized from recent progress in the optical detection, characterization, and manipulation of single quantum objects and may be exploited for producing intensity-squeezed light^92^.

The significance of the photon blockade effect is mainly motivated by the considerable applications of single-photons to the foundations of quantum theory as well as in quantum information science. It ensures a reliable single-photon source which is an essential ingredient for quantum simulation, and optomechanical system as a quantum non-linear device^93^. We show that the nonclassical features of photons,i.e., photon antibunching and blockade effects are limited by the thermal noise. Therefore, to ensure the realization of the photon blockade, and provide single-photon sources the thermal noise needs to be suppressed^94^.

#### Magnon statistics

**Figure 5:**
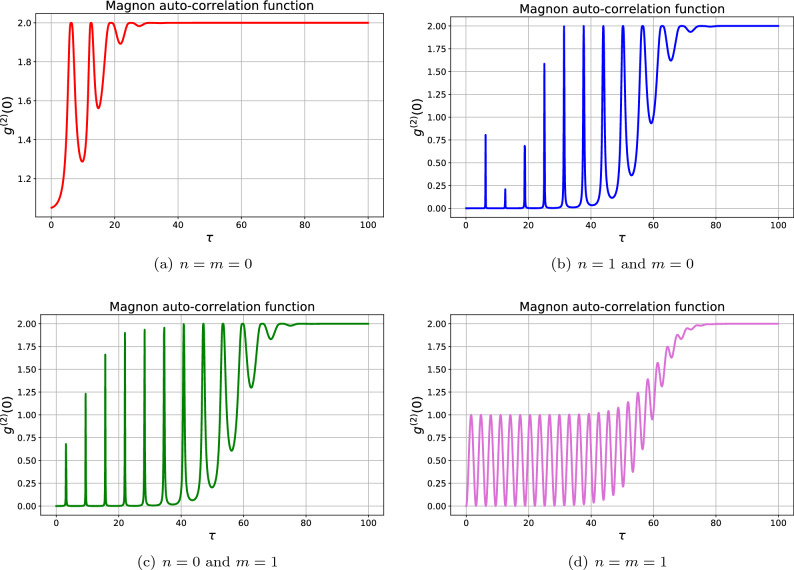
The effect of initial particles’ numbers on the time evolution of the magnon auto-correlation function for $E_b=0.1 \Gamma$, $G=5\Gamma$. The other parameters are the same as in Fig. 3. The results demonstrate both magnon antibunching and magnon blockade as the desired nonclassical features.

Now, we want to evaluate the time evolution of the magnon auto-correlation function. Let us start with the effect of the number of particles on the magnon statistics as depicted in Fig. 5. When the system is initiated with vacuum state $\left| n,m\right\rangle =\left| 0,0\right\rangle$ which implies that there is no photon in the WGM *b* and no magnon in the onset of interaction. The results show that the generated magnons do not obtain nonclassical properties as can be found in Fig. 5a. In this case, the magnons always are classical particles. When the system is initiated with only one photon in the WGM *b*, i.e., $\left| n,m\right\rangle =\left| 1,0\right\rangle$, the magnon antibunching may be realized for a relatively long time as is seen in Fig. 5b. Indeed, the photon in the WGM is annihilated and a magnon is created due to the scattering process. Also, the stability of nonclassical features, e.g., the magnon antibunching and blockade effects can be observed after the beginning of the interaction. As time passes, the number of magnons increases and they lose their nonclassical features (the bunched magnons may be realized) due to their interaction with the thermal environment. In this case, the magnons undergo some fast quantum-classical transitions during the interaction, i.e., $g_m^{(2)}(0)<1 \rightarrow g_m^{(2)}(0)>1$. Let us now consider the case in which the system is initiated with just one magnon, i.e., $\left| n,m\right\rangle =\left| 0,1\right\rangle$ which implies there is no photon in the WGM *b*. Generally, the patterns of magnon statistics in both Figs. 5b,c are similar, however, the quantum-classical transitions start sooner in the latter case. When the system is initiated with one photon and one magnon, the results are more interesting. Figure 5d reveals that the magnons statistics oscillate between sub-Poissonian (antibunched magnons) and Poissonian (coherent magnons) for a relatively long time, but finally, the magnons become thermal particles, i.e., $g_m^{(2)}(0)=2$. Generally, we can state that the magnon statistics strongly depends on the number of initial particle in the system, especially before approaching the steady state. As is expected the classical magnons prevail in the steady state regime because the thermal environment imposes its feature on the whole system.

**Figure 6:**
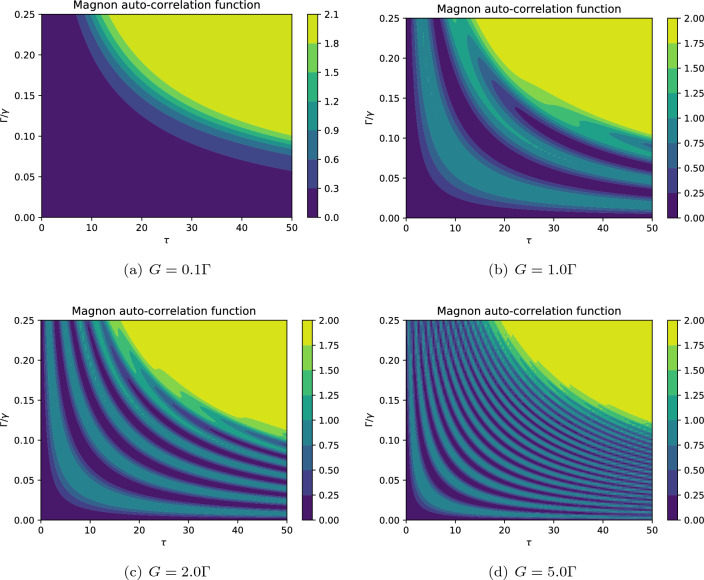
The effect of photon-magnon coupling constant on the time evolution of magnon statistics for $n=1$, $m=1$, and $E_b=0.1 \Gamma$. Note that $g_m^{(2)}(0) < 1$ implies the magnon antibunching. Also, the phenomenon of magnon blockade is realized when $g_m^{(2)}(0) \rightarrow 0$.

The time evolution of magnon statistics in the weak and strong coupling regimes is studied in Fig. 6. In the weak coupling regime where the enhanced optomagnonic coupling rate is much smaller than the decay rate of the WGM, i.e., $G \ll \kappa _b=\Gamma$, the results show that the nonclassical magnons’ features such as antibunching and blockade are seen in a relatively large window as shown in Fig. 6a. However, the magnons gradually become thermalized with their environment such that the bunched magnons remain in the steady state regime. Let us gradually increase the coupling strength to follow its influence on the magnon statistics. In Fig. 6b which is plotted for $G=1.0 \Gamma$, one can see that the magnons statistics shows an oscillatory manner in such a way that the magnons lose and re-acquire their nonclassical features several times after the onset of interaction. However, they finally become classical particles due to the presence of the thermal environment. Interestingly, the comparison between the minimum values of $g_m^{(2)}(0)$ in Fig. 6a,b reveals that the magnon blockade becomes more probable in the moderate coupling regime, e.g., $G=1.0 \Gamma$. Further increasing the amount of coupling strength demonstrates that the magnon statistics undergoes faster oscillations. Indeed, the quantum-classical transitions become faster by increasing the effective coupling strength *G* such that both classical and nonclassical magnons can be periodically realized during the interaction. Besides, it should be noted that the classical magnons are seen in larger time intervals by increasing the coupling strength. In fact, the magnon antibunching and magnon blockade gradually disappear in relatively strong coupling regime $G \gg \Gamma$ and instead coherent magnons appear during the interaction which results in the transition from Poissonian to super-Poissonian statistics, i.e., $g_m^{(2)}(0)<1 \rightarrow g_m^{(2)}(0) \simeq 1$. Note that all cases demonstrate a relatively large window in which the magnons possess thermal properties, especially for large damping rates and near the steady state regime.

It is worthwhile noting that a closer look at Fig. 3a,c reveals that it is possible to observe the perfect photon and magnon blockade phenomena, simultaneously, due to the fact that $g_{b(m)}^{(2)}(0) \rightarrow 0$ can be obtained at the same relatively long time interval.

In quantum science and technology, the study of nonclassical states of magnons is meaningful for the integration of magnons with other quantum platforms. Nonclassical magnons have attracted the interests of very diverse communities, from spintronics to quantum optics, from magnonics to CV quantum information, from condensed matter physics to astrophysics, from theorists to experimentalists and engineers^95^. Also, the coherent magnons allow the design of interference-based spin-wave devices and facilitate the implementation of wave-based computing devices. Some novel coherent states of matter, such as magnon Bose-Einstein condensates, provide a broad range of additional applications^96^. Besides, the thermally excited magnons have been intensively investigated thanks to their potential in computing devices and thermoelectric conversion technologies. A thermal magnon current mediated by coherent magnons via nitrogen-vacancy spin states may be exploited for the implementation of a device platform that hybridizes spin caloritronics and spin qubits^97^.

Furthermore, the magnon blockade as a pure quantum phenomenon opens up a pathway for designing a single magnon emitter^78,98^. The implementation and manipulation of single magnon sources will help to develop novel technologies with significant practical relevance for precision metrology, quantum information processing, and quantum simulation^59^.

#### Magnon-photon statistics: cross-correlation

**Figure 7:**
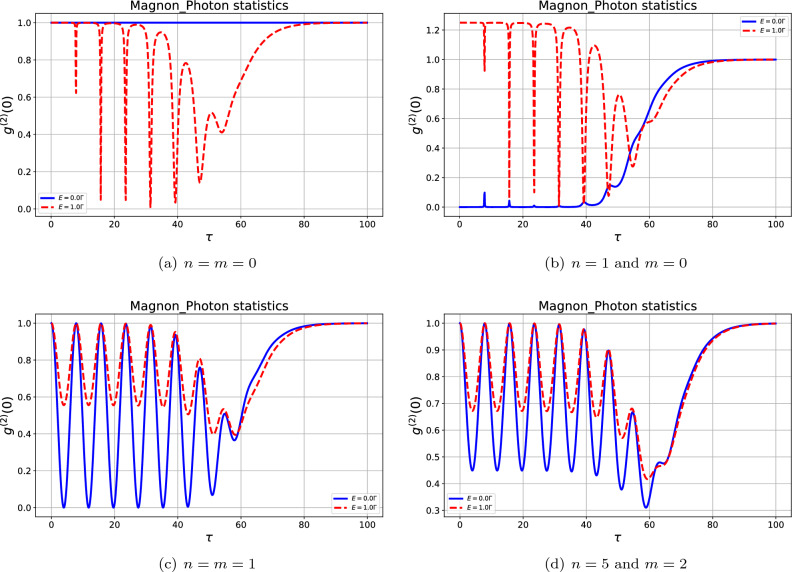
The time evolution of photon-magnon cross-correlation function for different initial particle numbers and driving field strengths with $G=2\Gamma$. The phenomenon of strong anti-correlation can be found in all plots, i.e., $g_{mb}^{(2)}(0) < 1$.

Now, we study the dynamics of the cross-correlation between photons and magnons. Figure 7 shows that the initial values of the cross-correlation strongly depend on the number of initial particles in the system. However, as time goes on this quantity undergoes an irregular oscillatory manner for a relatively long time and finally reaches its steady state. The oscillatory manner can be referred to the exchange of excitations between the magnons and photons. Generally, we can state that after the onset of interaction, the photons and magnons acquire nonclassical features, anti-correlation, i.e., $g_{mb}^{(2)}(0)< 1$, but then they gradually lose their anti-correlation and finally become uncorrelated in the steady state regime, i.e., $g_{mb}^{(2)}(0)= 1$. Let us take a look at each plot of Fig. 7. When the system is initiated with vacuum state $n=m=0$, the initial cross-correlation reads as $g_{mb}^{(2)}(0)= 1$ and the driving field governs the behavior of photon-magnon correlation as depicted in Fig. 7a. Especially for $E_b=0$, the system components always remain uncorrelated because there is no driving field to generate photonic or magnonic excitation, while the presence of the driving field, i.e., $E_b=1.0 \Gamma$, results in the anti-correlated particles. Besides, the phenomenon of perfect (strong) anti-correlation is expected in some moments of time, i.e., $g_{mb}^{(2)}(0) \rightarrow 0$. If the system is initiated with one photon in the WGM *b*, i.e., $n=1$ and $m=0$ as shown in Fig. 7b, the amount of initial cross-correlation reads as $g_{mb}^{(2)}(0)= 0$ which confirms the initial anti-correlation in the system in the absence of the driving field $E_b=0$. As time goes further, the magnons may be generated due to the scattering process. Therefore, the photon-magnon anti-correlation appears, however, the two modes eventually become uncorrelated as a result of the dissipation process. By contrast in the presence of the driving field, i.e., $E_b=1.0 \Gamma$, the system shows initial correlation, i.e., $g_{mb}^{(2)}(0) > 1$. Nevertheless, the photon-magnon correlation disappears and anti-correlated particles are generated which recalls a classical-quantum phase transition, i.e., $g_{mb}^{(2)}(0) > 1 \rightarrow g_{mb}^{(2)}(0) \le 1$. Let us consider the case in which the system initially contains equal numbers of photons and magnons, i.e., $n=m=1$. As can be seen in Fig. 7c, the system quantum statistics undergoes the same behavior in the absence and presence of the driving field. However, the amplitude of cross-correlation is considerably decreased in the presence of the driving field. Indeed, the perfect photon-magnon blockade may take place in some moments of time in the absence of the driving field. Note that in this case the photon and magnon are uncorrelated in the onset of interaction and steady state regime. Now, let us increase the number of initial particles, e.g., $n=5$ and $m=2$, and investigate the dynamics of cross-correlation. The same patterns as in Fig. 7c are seen in Fig. 7d. However, it should be noted that the amplitude of cross-correlation considerably decreases with respect to the latter case. Therefore, the number of initial particles limits the strength of the anti-correlation and blockade effect.

**Figure 8:**
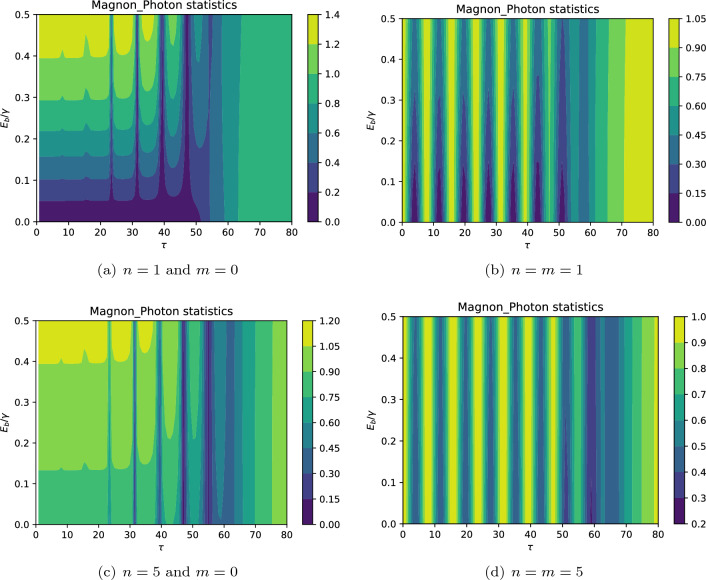
The contour plots of cross-correlation between photons and magnons for different initial particle numbers with $E_b=0.1 \Gamma$, and $G=5\Gamma$. The results show that the system may generate the correlated ($g_{mb}^{(2)}(0) > 1$), uncorrelated ($g_{mb}^{(2)}(0) = 1$), and anti-correlated particles ($g_{mb}^{(2)}(0) < 1$). Indeed, the system undergoes the quantum-classical transitions.

Now let us survey the 2D contours of the cross-correlation function of the system. Figure 8 shows the effect of particle number and the driving field strength on the time evolution of hybrid magnon-photon statistics. The periodic manner of cross-correlation is clearly seen in all plots. Also, both the number of particles, as well as the driving field, strongly affect the particles’ quantum statistics. Let us begin with the case in which the system is initiated by one photon in the WGM *b*. Figure 8a shows that after the beginning of interaction and for the relatively weak driving field, the system demonstrates strong anti-correlation between photons and magnons $g_{mb}^{(2)}(0) \simeq 0$. However, the particles become uncorrelated as time passes due to the effect of the dissipation process. Note that there is no initial magnon in the system, however, by increasing the strength of the driving field and thus the generation of magnons due to the scattering process, the uncorrelated magnons and photons gradually appear $g_{mb}^{(2)}(0) \simeq 1$ by applying the moderate driving fields. By further increasing the strength of the driving field, the particles may obtain correlation $g_{mb}^{(2)}(0) > 1$. It should be mentioned that the transitions from anti-correlated to uncorrelated and finally correlated particles can be controlled via tuning the strength of the driving field, especially for a relatively long time interval after the onset of interaction, i.e., $\tau <50$, but as time passes the particles become uncorrelated disregarding the strength of the driving field. Such a behavior can be found in Fig. 8c wherein the system is initiated with a larger number of photons, i.e., $n=5$ and $m=0$. However, in the latter case, there is no strong anti-correlation between the particles. Indeed, the anti-correlated particles are generated after passing enough time, especially, in the time interval $\tau \in (45,55)$. Let us consider the cases in which the system is initialized with equal numbers of photons and magnons. Both Fig. 8b,d reveal that the particles are uncorrelated at the beginning of the interaction, i.e., $g_{mb}^{(2)}(0) \simeq 1$. Besides, the transition from correlation to anti-correlation takes place in a regular oscillatory manner. The major difference between these cases is that in Fig. 8b the particles demonstrate stronger anti-correlation with respect to Fig. 8d.

Finally, it should be noted that when the system is pumped with a weak enough driving field, the system can not provide photon-magnon correlation as seen, particularly, in the right plots and generally in all plots of Fig. 8 because we always arrive at $g_{mb}^{(2)}(0) \lesssim 1$. In contrast, the generation of the anti-correlated particles is more plausible when the system is initiated with a weak enough driving field. More interestingly, the right plots of Fig. 8 clearly show that the driving field does not considerably influence the cross-correlation between the photon and magnons when the interaction begins with equal numbers of photons and magnons.

Physically, anti-correlation, i.e., $g_{mb}^{(2)}(0) < 1$ manifests clearly that both photon and magnons release their energies in the form of antibunched magnon-photon pairs. Besides, $g_{mb}^{(2)}(0) \rightarrow 0$ indicates that our considered system approaches a perfect photon-magnon emitter.

Finally, it should be emphasized that the interpretation of the auto-correlation function, i.e., $g^{(2)}(0)$ as a witness of successfully heralding a magnon Fock state depends on the temperature of the magnon mode as well as the mean number of thermal magnons. An important part of engineering quantum devices is quantum state preparation. The generation of nonclassical magnons allows the realizations of single-magnon detectors which are important to make use of magnonic states for quantum information processing^95^. Bittencourt et al. proposed a heralding protocol based on cavity optomagnonics in which a magnon Fock state is created by the measurement of an optical photon. Indeed, preparing Fock states in such platforms is an essential step toward the implementation of quantum information schemes. Also, the cross-correlation function can be used to detect the generation of photonic and magnonic Fock states^71^.

## Photon-magnon entanglement

Recalling that the magnons may be coupled with the optical photons through optomagnonic interaction to generate photon-magnon entanglement^99^. As mentioned, the YIG sphere supports two optical modes, namely the WGMs and one magnon mode. The two optical modes are driven by two laser pulses, respectively. The photon-magnon entanglement may be realized between the magnon mode and one of the two optical modes by applying the first pulse, and the state of the magnon mode is subsequently mapped into another optical mode via the second pulse^100^. Physically, the magnons can interact coherently with microwave photons via magnetic dipole interaction^101^. Moreover, the magnons and phonons may be coupled via the magnetostrictive interaction. The strong coupling between magnons and microwave photons has been demonstrated experimentally with magnetic YIG sphere^1,10^. This coupling not only facilitates magnon-photon entanglement^100^, but also allow an effective interaction between magnons and superconducting qubit^36^.

Now, we examine the photon-magnon entanglement as a measure of nonclassicality in our hybrid optomagnonic system. Some criteria have been proposed to determine the sufficient conditions for the characterization of intermodal entanglement^102–104^. Here, we restrict ourselves to one of these criteria which may be successfully used in detecting intermodal entanglement in bosonic systems, e.g., the photon-magnon entanglement. The inseparability criterion introduced by Hillary and Zubairy reads as

35 $$\begin{aligned} \mathcal {E}=\left\langle \hat{b}^\dagger (t) \hat{b}(t)\right\rangle \left\langle \hat{m}^\dagger (t) \hat{m}(t)\right\rangle - |\left\langle \hat{b}(t) \hat{m}(t)\right\rangle |^2. \end{aligned}$$

The negative value of the quantity $\mathcal {E}$ gives us the signature of the intermodal photon-magnon entanglement^102,105^. In what follows, we evaluate the photon-magnon entanglement by considering a two-mode cat state as the initial state of the system. The Schrödinger cat states not only play a key role in distinguishing the difference between classical and quantum physics^106^, but also are an indispensable resource for quantum computing^107,108^, quantum teleportation^109^ and high-precision measurements^110^. Under a strong driving field, e.g., microwaves, the magnons will reach a coherent state, where the mean magnon number is equal to its variance^95^.

Magnon cat state as a macroscopic quantum superposition of collective magnetic excitations not only provides fundamental tests of macroscopic quantum effects but also allows quantum metrology and quantum computation. Interestingly, both even and odd magnonic cat states can be remotely generated by performing local non-Gaussian operations on the optical mode that is entangled with magnon mode through pulsed optomagnonic interaction^99^. Besides, the magnonic cat states could be prepared by pulsed sideband driving, in the optical domain. Recently, the analog quantum control of magnonic cat states by a superconducting qubit was reported in^111^. So, it is worthwhile to investigate the photon-magnon entanglement when the system initiated with even and odd Schrödinger cat states denoted by $\left| \Psi (\alpha )\right\rangle _{+}$ and $\left| \Psi (\alpha )\right\rangle _{-}$, respectively. These states are defined below

36 $$\begin{aligned} \left| \Psi (\alpha )\right\rangle _{\pm }= & {} \frac{1}{\sqrt{2(1 \pm e^{-2\alpha ^2})}}(\left| \alpha \right\rangle \pm \left| -\alpha \right\rangle ), \\ \nonumber \left| \beta \right\rangle= & {} e^{-\frac{|\beta |^2}{2}} \sum _{l=0}^{\infty } \frac{\beta ^l}{l!} \left| l\right\rangle , \qquad \beta =\pm \alpha , \end{aligned}$$

where we assume that $\alpha$ takes real values for convenience. It should be noted that the odd (even) magnon cat state can be remotely generated by performing a single-photon subtraction (addition) operation. Indeed, the initial magnon coherent state turns into a non-Gaussian state by applying a single-photon operation on the optical mode. After a projective measurement on the optical mode, the magnon state collapses into a Schrödinger cat state^99^.

These states possess some interesting features. For instance, the even cat states show super-Poissonian (classical) statistics and provide squeezing properties. In contrast, the odd cat states present sub-Poissonians (nonclassical) statistics and no squeezing evidence. The reversal role between the even and odd coherent states regarding quadrature squeezing and sub-Poissonian statistics can be readily checked. Also, the even and odd cat states result in positive and negative amplitude at the symmetry center in the Wigner functions^99^. All these characteristics may rise due to the difference in parity of even and odd cat states. In practice, it is possible to prepare different magnon cat states by choosing suitable single-photon operations.

**Figure 9:**
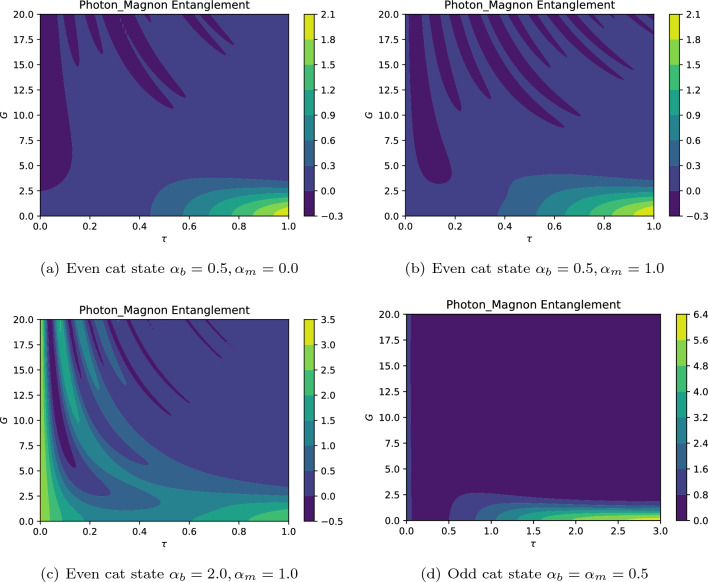
The photon-magnon entanglement dynamics against $\tau =\Gamma t$ for $\Gamma =1.4 \gamma$, $E_b=0.1\gamma$ ($P_d \simeq 0.037 \mu$W). The regions with negative values indicate photon-magnon entanglement.

Besides, the generation of the optical Schrödinger cat states has been both theoretically and experimentally demonstrated using homodyne detection and photon number states as resources^112^. Both even and odd Schröndiger cat states have been successfully generated experimentally in various physical systems such as optic system^112,113^, superconducting quantum inference device^114^, and trapped ion system^115^. Considering these Schrödinger cat states and using Eq. (35), we arrive at

37 $$\begin{aligned} \mathcal {E}_{\pm }= & {} \bigg (e^{-2\Gamma t} \big [ (\mathcal {N}_{\pm } (\alpha _b) +E_b^2) \cos ^2 Gt + \mathcal {N}_{\pm }(\beta _m) \sin ^2 Gt \big ] + \left\langle \hat{\mathcal {B}}^\dagger _{\text{B}}(t)\hat{\mathcal {B}}_{\text{B}}(t)\right\rangle + \left\langle \hat{\mathcal {M}}^\dagger _{\text{B}}(t)\hat{\mathcal {M}}_{\text{B}}(t)\right\rangle \bigg ) \\ \nonumber\times & {} \, \bigg ( e^{-2\Gamma t} \big [ \mathcal {N}_{\pm }(\beta _m) \cos ^2(Gt) + (\mathcal {N}_{\pm } (\alpha _b) +E_b^2) \sin ^2(Gt) \big ] + \left\langle \hat{\mathcal {B}}^\dagger _{\text{m}}(t)\hat{\mathcal {B}}_{\text{m}}(t)\right\rangle + \left\langle \hat{\mathcal {M}}^\dagger _{\text{m}}(t)\hat{\mathcal {M}}_{\text{m}}(t)\right\rangle \bigg ) \\ \nonumber{} & {} - \, e^{-4\Gamma t} \left( \alpha _b^2+E_b^2+\alpha _m^2 \right) ^2 \sin ^2 Gt \cos ^2 Gt \end{aligned}$$

where

38 $$\begin{aligned} \mathcal {N}_{\pm } (\alpha _k) = \alpha _k^2 \frac{1\mp \exp { \left( -2\alpha _k^2 \right) }}{1\pm \exp { \left( -2\alpha _k^2 \right) }}, \quad k=b,m, \end{aligned}$$

where $\alpha _b$ and $\alpha _m$ stand for the intensities of the initial coherent photons and magnons, respectively. Now, let us numerically investigate the photon-magnon entanglement. Figure 9 shows the dynamics of photon-magnon entanglement criterion for both even and odd coherent cat states in a long-range coupling strength *G*. The first three plots correspond to the even cat state, e.g., $\left| \Psi (\alpha _b),\Psi (\alpha _m)\right\rangle _{+}$ with different initial intensities, while the last plot is obtained when the system is initiated with the odd cat states $\left| \Psi (\alpha _b),\Psi (\alpha _m)\right\rangle _{-}$. For the considered conditions, the entanglement can be only observed with even cat states. However, it should be noticed that the dynamics of entanglement depends on the initial particle intensities as well as the photon-magnon coupling strength. For instance, Fig. 9a shows that the entanglement exists between photons and magnons for $G>2.2$ just after the onset of interaction. By increasing the mean number of initial particles, one can observe that the photons and magnons are initially separable, but fastly become entangled after the onset of interaction as can be found in Fig. 9b,c. We can state that by increasing the intensity of cat states, the particles obtain further classical properties and therefore the system loses its entanglement, particularly in the relatively weak interaction regime. On the other hand, when the system is initialized with odd cat state, there is no entanglement between the photons and magnons. Generally, our results show that the intermodal entanglement may be observed in the strong coupling regime, especially when the system is initiated with lower intensity (population) even cat states. Furthermore, the numerical analyses show that there is no entanglement between photons and magnons when the system is initialized with odd cat states. In fact, the parity of Schrödinger cat states may influence the dynamics of photon-magnon entanglement. Since we evaluate the photon-magnon entanglement criterion for both even and odd coherent states with the same parameters, therefore, the odd parity of initial photons and magnons may result in no photon-magnon entanglement. Also, the state $\left| \Psi (\alpha )\right\rangle _+$ ($\left| \Psi (\alpha )\right\rangle _-$) contains only even (odd) energy eigenfunctions, due to the quantum interference between the two coherent states $\left| \pm \alpha \right\rangle$^116^. So, the photons and magnons may become entangled or disentangled due to the quantum interference between their wave pockets. The realization of Schrödinger cat superposition was verified by the detection of the quantum mechanical interference between the localized wave packets. Interestingly, it was shown that the vanishing interference signal is a signature of an odd Schrödinger cat state^115^. Also, the entanglement-interference complementarity relation was experimentally demonstrated, particularly, via a superconducting circuit. Both the interference and the entanglement originate from the original coherence of the interfering system. In contrast, the coherence, as a resource can be converted to entanglement or used for interference^117^.

**Figure 10:**
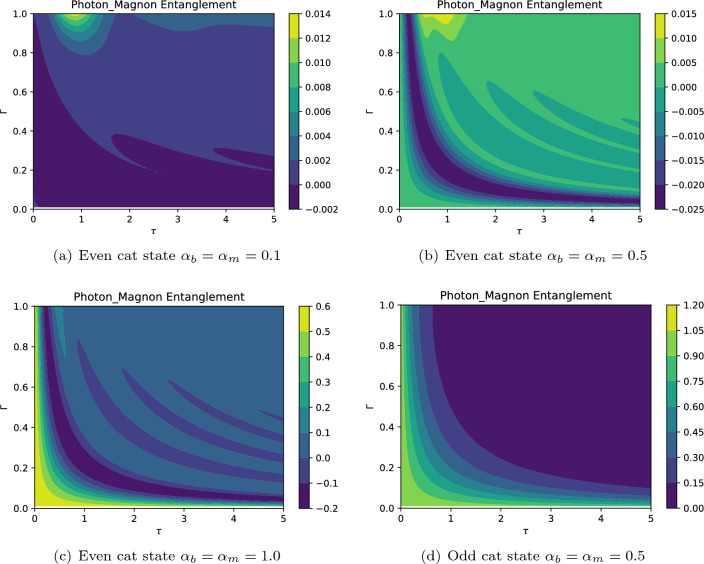
The photon-magnon entanglement dynamics against $\tau =\Gamma t$ for $G=3.2 \gamma$ and $E_b=0.1\gamma$ ($P_d \simeq 0.037 \mu$W). The negative values of this quantity indicate that the hybrid optomagnonic state is entangled.

Also, the effect of the dissipation rate on the entanglement dynamics shows the same behavior as the coupling strength. The results presented in Fig. 10 confirm that the entanglement may be observed when the system is initiated with even cat states. Also, when the system initially contains a larger number of particles, there is no initial intermodal entanglement, but the components of the system become entangled after the onset of interaction. Generally, as may be expected, the entanglement experiences a decaying behavior as time goes on. It is worth mentioning that the phenomenon of disentanglement may stem from the dissipative process, independent of the coupling with the environment, and is sensitive to the initial conditions^118^. Besides, a rough comparison between Fig. 10a,c reveals that for even cat states with lower initial mean particle number, the entangled state may be generated in a larger window as shown in Fig. 10a. Once again, we see that the system with initial odd cat states shows no photon-magnon entanglement. Note that the energy transfer may affect the dynamics of entanglement. It was suggested that the concurrence as a measure of entanglement may be expressed as a function of energy transfer. Hence, one can define a critical energy *Ec* such that below which $(E<E_c)$ the system must be non-entangled, and above which $(E>E_c)$ the system must be entangled^118^. Consequently, depending on the chosen initial state of the system, e.g., the even and odd Schrödinger cat states, the energy transfer between photons and magnons may result in the non-entangled states. However, it should be noted that finding the prpoer critical energy may be complicated due to its dependence on the state of system that prevents further investigations^118^. Finally, it should be emphasized that we have here used one of the criteria introduced by Hillery and Zubairy to detect intermodel entanglement by considering initial coherent cat states. Further investigations may demonstrate intermodel entanglement when the system is initiated with other possible physical conditions, e.g., by considering different initial states for the system and interaction regimes.

## Experimental feasibility and applications

As mentioned, Osada et al. experimentally implemented a system of cavity optomagnonics where a sphere of ferromagnetic material supports WGMs for photons and the magnetostatic mode for magnons. They utilized the WGM resonator with a 750 $\upmu$m diameter YIG sphere which is highly transparent at the optical wavelength of 1.5 $\upmu$m and has a refractive index of 2.19. The light source with wavelength 1.5 $\upmu$m originated from an external-cavity diode laser is introduced through a fiber polarization controller and then coupled to the WGM resonator via a tapered silica optical nanofiber. Further details can be found in^4^.

In such a system, the WGM photons are scattered by the GHz magnons. Indeed, the high-quality WGM cavity drastically enhances magnon-induced BLS. The asymmetry nature of the BLS allows one to govern and manipulate the Stokes or anti-Stokes scattering events^74^. One can create or annihilate magnons in a highly controlled manner by changing the polarization of the input laser^4^. The BLS is a well-established technique to study and manipulate magnons. Such a mechanism has been adopted for preparing nonclassical states of magnons^55,74^.

Quantum information and its resources such as squeezing and entanglement have been pursued for discrete variables (DV), and continuous-variables (CV) systems. The squeezed and entangled photons or magnons demonstrate sub-shot noise and are fruitful for quantum teleportation^119,120^. The generation of CV nonclassical states paves the way for future quantum computation protocols^121^. Notably, CV quantum information gives us more opportunities than DV formalism due to the larger accessible Hilbert space^122^.

Although, the generation and coherent control of quantum states in a macroscopic spin system still remains an outstanding challenge, the nonclassical states of the magnon, including the single-magnon state and the equal amplitude superposition state of a single magnon and vacuum has been deterministically generated and benchmarked^123^. Indeed, the precise and deterministic quantum control of a single-magnon introduces the YIG spin system as one of the largest quantum systems that can generate macroscopic quantum states. The authors in^123^ explored the possibility of utilizing quantum states of the magnon in a ferrimagnetic YIG system to implement quantum information processing. Besides, the quantum control of a single-magnon enables us to explore promising applications in quantum engineering such as the quantum transducer^124^ and quantum network^125^. The hybrid optomechanical-magnetic system may be used as a quantum device. Hence, the single-excitation level and the simultaneous blockade of a photon, phonon, and magnon are of fundamental interest and deserve further investigations. As a pure quantum phenomenon, the magnon blockade manifests some intriguing quantum properties and its manipulation is necessary for the preparation of single-magnon sources^126^.

The intermodal magnon-photon entanglement is an interesting quantum resource because of its fundamental importance to understand macroscopic quantum phenomena and the classical-quantum transition in magnetic systems, and their potential applications in CV quantum information. For instance, the photon-magnon entanglement can be realized by activating the optomagnonic and optomechanical anti-Stokes processes, which transfer the magnonic and mechanical states to their respective anti-Stokes photons. Interestingly, the magnon state can be read out by using the anti-Stokes process of the BLS^74^. Also, the magnon cat states that allow fundamental tests of macroscopic quantum effects are promising in quantum metrology and quantum computation. The magnon-photon entanglement can be used for the remote generation of magnon Schrödinger cat states^99^.

Briefly, our results address how to generate the nonclassical states in a hybrid optomagnonic system and pave the way to explore its promising applications in quantum information processing and quantum engineering. Finally, it is worth mentioning that a hybrid optomagnonics system provides a promising platform to study both fundamental quantum physics and fruitful applications such as quantum transducers, quantum memories, high precision measurements, and logic gates^95^. Even though some of the proposals have been experimentally benchmarked, the state-of-art design of such systems needs further investigation. Developing the current proposals to the quantum limit may result in new physics and present various paths towards interesting phenomena and novel technologies.

## Summary and discussion

We propose a theoretical model to study the nonclassical features of a hybrid optomagnonic system under the influence of the anti-Stokes scattering process. Indeed, we assume a YIG sphere that simultaneously supports a magnetostatic mode of magnons and two WGMs of optical photons. The system undergoes the magnon-induced BLS, i.e., the photons in the WGMs are scattered by lower-frequency magnons. Consequently, the sideband photons are generated such that their frequency is shifted by the magnons’ frequency. The verified sideband asymmetry in magnon-induced BLS facilitates the selective creation or annihilation of magnons in the Kittel mode with optical photons^4^. These unique features of the system allow it to serve as an interesting test bed for investigating diverse quantum contexts, including quantum optics and spintronics.

To achieve our goal, based on the context of the open quantum system, we derive the analytical expressions of the time-dependent operators corresponding to the photonic and magnonic subsystems and then proceed to deal with the nonclassical features of the photons and magnons. In particular, we investigate the auto-correlation and cross-correlation among the components of the system as well as their intermodal entanglement.

Generally, the results show that the particles, e.g., both photons and magnons acquire nonclassical characteristics such as antibunching, anti-correlation, and blockade phenomena. Also, the phenomenon of simultaneous photon-magnon blockade may take place during the interaction. Besides, the system provides the potential for tracking the quantum-classical transition because the particles present sub-Poissonian (pure quantum), Poissonian (semi quantum-classical; coherent), and super-Poisssonian (pure classical) statistics, particularly, before approaching the steady state regime. However, it should be mentioned that both photons and magnons lose their nonclassical features and become thermal particles due to their interaction with thermal environments. Although the nonclassical features eventually disappear due to the dissipation process, we demonstrate that the system can maintain strong antibunching and anti-correlation for a relatively long time before the thermalization.

It is noteworthy that the nonclassical features of the particles may be manipulated by tuning the system and bath parameters. In particular, the system obtains strong antibunching and anti-correlation in the presence of the weak driving field. Also, the nonclassical characteristics may be empowered if the system is initiated with small numbers of particles. For instance, when the system is initialized with just one particle (photon or magnon), both antibunching and anti-correlation may be realized for a relatively long time. Furthermore, the results reveal that the coupling strength between photons and magnons strongly affects the quantum statistics of the particles. For instance, the magnons gradually lose their antibunching effects in the weak coupling regime, but they periodically lose and re-obtain antibunching in the moderate coupling regime. Note that the oscillatory manner of the magnon statistics maintains even in the strong coupling regime, however, the magnons mostly acquire coherent properties in this regime. In other words, the photon-magnon coupling strength may be used to control the period of the quantum-classical transitions.

Finally, it should be noted that the unique nonclassical features of such a system, e.g., antibunching and anti-correlation, may provide interesting applications at the crossroad between quantum optics and spintronics.

On the other hand, the intermodal photon-magnon entanglement may be observed in the strong coupling regime, especially when the system is initiated with the even cat states. Although the dissipation process plagues the intermodal entanglement, the entangled optomagnonic states may be generated in a larger window with lower initial particle mean numbers. Briefly, our model offers a promising vision for the realization of realistic hybrid optomagnonic systems, and finds potential applications in quantum information processing based on the generation of single-particle sources.

### Supplementary Information

Supplementary Information.

## Data Availability

The datasets generated and/or analyzed during the current study are available from the corresponding author upon reasonable request.
