# Supplementary material for: Dissipative dynamics of optomagnonic nonclassical features via anti-Stokes optical pulses: squeezing, blockade, anti-correlation, and entanglement
Source: Sci Rep. 2023 Aug 7;13:12757. doi: 10.1038/s41598-023-39822-y (PMC10406899; doi:10.1038/s41598-023-39822-y)
Supplement: Supplementary file 1 — Supplementary Information. [file 41598_2023_39822_MOESM1_ESM.pdf]

Appendix

Using Eqs. (13) and (16), we proceed to obtain the expectation value of the bath operator.

$$\hat{\mathcal{B}}_{\text{ab}}(t) = -i\sum_k g_k b_k(0)B(t), \tag{1}$$

where

$$\begin{aligned} B(t) &= \frac{e^{-(i\omega+\Gamma+iG)t}}{2[i\omega_k-(i\omega+\Gamma+iG)]}+\frac{e^{-(i\omega+\Gamma-iG)t}}{2[i\omega_k-(i\omega+\Gamma-iG)]} \\ &+ \frac{(i\omega+\Gamma-i\omega_k)e^{-i\omega_k t}}{(i\omega+\Gamma+iG-i\omega_k)(i\omega+\Gamma-iG-i\omega_k)}, \end{aligned} \tag{2}$$

$$\langle\hat{\mathcal{B}}^{\dagger}_{\text{ab}}(t)\hat{\mathcal{B}}_{\text{ab}}(t)\rangle = \sum_k\sum_{k'} g^*_k g_k \, \langle \hat{b}^{\dagger}_{k'}(0)\hat{b}_k(0)\rangle \, B^*(t)B(t), \tag{3}$$

which can be rewritten as below

$$\begin{aligned} \langle\hat{\mathcal{B}}^{\dagger}_{\text{ab}}(t)\hat{\mathcal{B}}_{\text{ab}}(t)\rangle &= \sum_k\sum_{k'}[g_k]^2 n_k(\omega_k)B^*(t,\omega_{k'})B(t,\omega_k)\delta_{kk'} \\ &= \int d\omega J(\omega)n(\omega)\chi^2(t), \end{aligned} \tag{4}$$

where in the last line, we have set $\omega_k \to \omega$ and replaced the summation by an integral. For a super-Ohmic spectral density function ($s = 3$) we have

$$J(\omega) = \eta\omega^3\omega_c^{-2}e^{-\omega/\omega_c}, \tag{5}$$

and also

$$\begin{aligned} n(\omega) &= \frac{1}{e^{\frac{\omega}{k_{\text{B}}T}}-1}, \\ \chi(t) &= \frac{-e^{-\Gamma t}[\Gamma\cos Gt - G\sin Gt]+\Gamma}{\Gamma^2+G^2}. \end{aligned} \tag{6}$$

Considering these relations, we arrive at

$$\begin{aligned} \langle\hat{\mathcal{B}}^{\dagger}_{\text{ab}}(t)\hat{\mathcal{B}}_{\text{ab}}(t)\rangle &= \eta\omega_c^{-2}\chi^2(t)\int d\omega\frac{\omega^3 e^{-\omega/\omega_c}}{e^{\frac{\omega}{k_{\text{B}}T}}-1}, \\ &= \eta\omega_c^{-2}\chi^2(t)(k_{\text{B}}T)^4\int dX\frac{X^3 e^{-X\frac{k_{\text{B}}T}{\omega_c}}}{e^X-1} \end{aligned} \tag{7}$$

where we have set $X = \frac{\omega}{k_{\text{B}}T}$. For $\omega_c = pk_{\text{B}}T$ (p is an integer), one arrives at

$$\langle\hat{\mathcal{B}}^{\dagger}_{\text{ab}}(t)\hat{\mathcal{B}}_{\text{ab}}(t)\rangle = \eta\omega_c^{-2}[\frac{-e^{-\Gamma t}[\Gamma\cos Gt - G\sin Gt]+\Gamma}{\Gamma^2+G^2}]^2(k_{\text{B}}T)^4\Psi\big(3,\frac{n+1}{n}\big) \tag{8}$$

where $\Psi(p, x)$ is the p-th polygamma function.
